# Case-based exercises fail to improve medical students' information management skills: a controlled trial

**DOI:** 10.1186/1472-6920-6-14

**Published:** 2006-03-01

**Authors:** Heidi S Chumley, Alison E Dobbie, John E Delzell

**Affiliations:** 1 Department of Family Medicine, University of Kansas School of Medicine, Kansas City, USA; 2 Department of Family and Community Medicine, University of Texas Southwestern Medical Center at Dallas, Dallas, USA

## Abstract

**Background:**

Tomorrow's physicians must learn to access, retrieve, integrate and apply current information into ambulatory patient encounters, yet few medical schools teach 'real time' information management.

**Methods:**

We compared two groups of clerkship students' information management skills using a standardized patient case. The intervention group participated in case-based discussions including exercises that required them to manage new information. The control group completed the same case discussions without information management exercises.

**Results:**

After five weeks, there was no significant difference between the control and intervention groups' scores on the standardized patient case. However, third rotation students significantly outperformed first rotation students.

**Conclusion:**

Case-based exercises to teach information management failed to improve students' performance on a standardized patient case. Increased number of clinical rotations was associated with improved performance.

## Background

When today's medical students graduate, they will conduct patient encounters using multiple technology-enhanced decision support systems. Current medical student training in ambulatory settings may not prepare students for this type of practice. Students often learn from physicians who generate few (0.01 to 0.8) clinical questions per patient encounter and infrequently use information technology to answer clinical questions at the point of care [1-3] Clinicians have previously reported that answering clinical questions is too time consuming to be practical during clinical sessions[4,5] However, that situation is changing. Improved information management tools, including personal digital assistants (PDAs) and Internet capable wireless computers, now allow rapid access to Web-based clinical information in ambulatory settings.

Medical students must develop information management skills as a routine, integral part of the ambulatory patient encounter. Information management skills include: asking and refining clinical questions; accessing, retrieving, integrating and applying information into a clinical situation; and managing the doctor/patient/technology interface. Managing information at the point of care requires different skills than traditional evidence-based medicine (EBM) as taught in most medical schools [6] In traditional EBM, the learner develops a clinical question, performs a literature search, selects and appraises an appropriate research study, and draws conclusions. The EBM process usually occurs remotely from the patient encounter and requires time, the ability to understand the source literature, and critical appraisal skills.

While EBM skills are important for medical learners, many clinical questions can be answered at the point of care without critical appraisal. For example, using an electronic drug database in a PDA to investigate potential drug interactions is information *access and retrieval*. Modifying the patient's medications to adjust for interactions is information *integration and application*. The learner accesses an information source he/she deems reliable, and finds the answer in approximately 20 seconds during the office visit.

Few studies have investigated students' ability to manage information in clinical settings. In a study by Bergus and colleagues, fourth year medical students evaluated a standardized patient (SP), then read an article about a diagnostic test relevant to the patient's presentation. Most students appraised the article correctly, but few could apply the information to the individual patient[7] In contrast, Webershock and colleagues demonstrated that, following an EBM seminar, third-year students could both appraise an article and integrate that information into a paper case[8] Davidson and colleagues conducted a more complex skills assessment by having SPs ask a question of third year medical students. Students then formulated a clinical question, performed a Medline search, selected and appraised a journal article and transmitted results to the patient. Students did well in this applied EBM exercise, averaging 3.7–4.0 on a scale of 1 (poor) to 5 (superior) for each task as evaluated by faculty and librarians[9] In summary, after a non-clinical EBM course, graduating students demonstrated EBM knowledge but had difficulty applying EBM information in the clinical setting. However, third year students applied EBM skills adequately to a paper case and standardized patient encounter given structured directions and sufficient time

A 2005 literature search yielded no previous studies investigating students' abilities to access, retrieve, integrate and apply information in real time patient encounters. In this study, we investigated whether case-based discussions with information management exercises improved students' information management skills as evaluated on a standardized patient case. We compared (1) intervention and control groups on two different clerkships and (2) first and third groups from both clerkships in academic year 2005–6.

## Methods

### Setting and subjects

The University of Kansas School of Medicine is a state medical school with 175 students per year and two clinical campuses. All 120 third year medical students on the Kansas City campus spend six weeks each in the Family Medicine (FM) and Ambulatory Medicine/Geriatrics (AM/G) clerkships. These clerkships are always adjacent. Students either complete six weeks of FM followed by six weeks of AM/G or vice versa. In our study, all 55 students from the 2005 fall semester completed the information-management SP case in week five of the 12-week FM and AM/G adjacent clerkships. The SP case was a required activity; however, students signed consents to include their results in the analysis. Our human subjects committee granted the study exempt status.

### Study design

In a prospective controlled group study of 55 third year students, we compared our intervention and control group performance on an information management SP case. Students who began the twelve weeks on FM formed the intervention group. Those who began on AM/G formed the control group. After six weeks, the students swapped clerkships. Students on both clerkships recorded similar numbers and types of ambulatory encounters in their patient logs. Neither clerkship offers any formal EBM curriculum. However, all students learned EBM principles (but not clinical information management) through longitudinal didactic sessions across their first two years.

### Educational intervention

The intervention group students participated in weekly case-based discussions with added exercises designed to teach students to access, retrieve, integrate and apply new information in medical-decision making. (For examples, see Table 1.) Intervention group students completed the information management exercises using their PDAs plus Internet capable wireless computers. The control group participated simultaneously in weekly discussions using the same cases and content (but with no emphasis on managing information), with their PDAs, but without tablet computers with Internet access.

### Evaluation: – The SP case

We used our institution's standard template to create an SP case designed to assess third year medical students' information management skills at the point of care. The SP was a healthy woman seeking travel advice for a trip to Botswana, Africa. During the SP encounter, students had the same access to the Internet and their PDAs as during the case based discussions and in the family medicine clinic. We expected students to *access *an appropriate Web site, *retrieve *information about travel medicine, *integrate *the appropriate questions into the encounter and *apply *the information by offering the recommended medications, immunizations and travel advice. Immediately after each encounter, the SP graded the student on a checklist developed using information from the Center for Disease Control's website. (See Table 2) We digitally recorded every encounter and the clinical skills laboratory staff audited 10% of the SP encounters to verify the SP's coding accuracy.

### Statistical considerations

Using a non-paired t-test, we compared performance on the SP case between (1) intervention group students (who had completed information training) and control group students (who had no information training) and (2) first and third clerkship groups in academic year 2005–6. Thirteen out of 23 checklist items required the use of technology. (See Table 3) For power analysis purposes, we estimated that students not using technology would average 10/23 (43%) and students using technology would average 18/23 (78%). (Our third year medical students' mean score across multiple validated cases is 75%). Using these estimates, we calculated a sample size of 15 per group to detect significance at the 0.05 level with a power of 0.9.

## Results

Fifty-five students (26 intervention and 29 control) completed the standardized patient case and 42 (19 intervention and 23 control) consented to have their information included in the analysis (76%). There was a difference between consenters and non-consenters on total scores. Students who consented scored lower than those who did not. (14.0, 15.5, p = 0.05)

Percentages of consenting students completing each checklist item are shown in Table 2. Ninety percent of students accessed the Internet and 62% used a PDA during the encounter. Ninety-eight percent of students offered medication for malaria prophylaxis. Forty-eight percent asked if the patient would be working with animals, but only 10% inquired if she would be providing health care. Although the SP questioned every student about medication interactions, only 38% confirmed a potential interaction between doxycycline and antacids and of these 17 students, 71% (12) discussed how to take the medication to avoid the interaction.

There was no difference in total scores between intervention and control group students (13.4, 14.4, p = 0.12). However, students in both the intervention and control groups on the third rotation scored significantly higher than first rotation students. (13.6, 15, p = 0.038). However, when we examined the 13 technology items, there were no differences between intervention and control group scores (7.5, 7.9, p = 0.25) or between first and third rotation group scores (7.3, 8.3, p = 0.08).

## Discussion

Intervention group students who received case-based training in information management scored no higher on the SP case than students who received no such training. Possible explanations include that the intervention did not adequately teach information management skills or that the SP case did not accurately evaluate those skills. Also, students may have failed to apply/transfer the information management skills from the case-base discussions to the SP experience because the learning context changed from paper cases to a mock clinical encounter. In addition, students do not learn the clinical content (travel medicine) during any third year rotation, so we were testing on a content area that we had not formally taught. However, we deliberately chose this content because we considered that the lack of formal teaching 'leveled the playing field' for finding travel information that is easily retrieved from the Internet. Third rotation students performed better than first rotation students on the total score, but not on the technology sub-score. Third rotation students' better performance may be explained by their increased clinical experience, or by the fact that they learned the case expectations from students in the previous groups. All groups' mean scores were lower than the usual average score of 75% seen across multiple validated SP cases in our setting.

All but one student *accessed *a reputable site (e.g. the Center for Disease Control) and *retrieved *travel information for Botswana, and most *integrated *and *applied *information pertaining to malaria prophylaxis (but not health care delivery or working with animals). Thus, if we group information management skills into two levels – *Level 1 *(*access and retrieval*) *a*nd *Level 2 *(*integration and application*), our students did demonstrate some skills on both levels. However, their performance on Level 2 skills (integration and application) was generally poor. Even in our best-performing group only 67% consulted their PDAs to investigate a potential drug interaction. Students' performance may have been adversely affected by the time pressure of a 20-minute encounter, although we consider this unlikely. On our SP case feedback form, we routinely question students on whether or not they had adequate time for the case. For most cases, 2–3 students per group report having insufficient time. For the travel case, no student reported having insufficient time.

Even when questioned directly about medication interactions, only 38% of all students consulted their PDAs to investigate a possible interaction. Most students wrongly informed the standardized patient that there was no potential interaction between doxycycline and antacids without consulting their PDA, a task that requires minimal time (20 seconds or less). Two plausible explanations for this lack of PDA use are that students did not know how to use the drug database program or they incorrectly assumed that they knew the answer. In our institution, all third year students are given PDAs and receive formal training on logging patient encounters, but they receive no formal training on using medical programs such as Epocrates^©^. Our experience with this highly Internet savvy generation is that most students quickly master the capabilities of their PDAs, but we have cannot report any empiric data on the numbers of students who could not operate the drug database versus those who chose not to consult it.

Our study has several limitations. We conducted it with a small number of learners, at a single institution, using a single SP case for evaluation. Global student competence cannot be reliably assessed by a single standardized patient case. However, in this pilot study, we were not evaluating individual students' global performance. Rather, we were measuring group performance on one particular skill set. We accounted for the suspected variation among student performance and the study had sufficient power to determine if one group of students performed better than another.

## Conclusion

Managing clinical information at the point of care is an important skill for future physicians, yet the literature is sparse on teaching information management in ambulatory settings. Unfortunately, our curricular intervention did not impact students' information management performance as measured by an SP case. Student performance did improve with increasing clinical experience. One important finding was that few students accessed an available PDA database to investigate potential drug interactions even when directly prompted by the SP. More concerning was the finding that most students indicated incorrectly that there was no potential interaction. Wrongly answering patients' questions without using readily available information systems is an important and preventable source of medical errors. Medical educators must teach learners to develop information management habits that reduce medical errors, including prescribing errors. At our institution, we will re-evaluate our teaching methods, with the specific aim of teaching students to check for medication interactions at every encounter. Future studies must investigate other educational interventions that demonstrate improvements in students' information management skills.

## Competing interests

The author(s) declare that they have no competing interests.

## Authors' contributions

HC conceived of the study, developed educational interventions and the standardized patient case, participated in its design and coordination, performed the statistical analysis, and helped write the manuscript.

AED participated in the conception and design of the study, educational interventions and standardized patient case, and helped to write the manuscript.

JD participated in the interpretation of data and helped to write the manuscript.

All authors read and approved the final manuscript.

## Pre-publication history

The pre-publication history for this paper can be accessed here:



## Figures and Tables

**Table 1:** A healthy 55-year-old woman traveling to Botswana presents for travel advice. Her health maintenance is current except for her tetanus booster.

**Case Details**	**Student Tasks**
Patient enquires about travel immunizations and prophylactic medications for Botswana	Students should: 1. access CDC Website (or similar) via Internet for current travel recommendations for Botswana 2. offer malaria prophylaxis 3. enquire about planned activities (e.g. providing health care) 4. provide information about food/water safety 5. advise re preventing mosquito bites
Patient indicates her preference for every day malaria prophylaxis	Students should access Web or PDA, and suggest doxycycline for malaria prophylaxis *
Patient asks if doxycycline will interfere with her antacids	Students should run an interaction check on their PDAs*

* NOTE: Students who delivered accurate information without using information resources received full credit.

**Table 2:** Information management exercises in the case-based discussions

**Case topic**	**Information Management Exercise**	
	
	**Access and retrieve**	**Integrate and apply**
Coronary Artery Disease	risk calculators (Society of General Internal Medicine)	select appropriate cardiac diagnostic studies
Chronic Obstructive Pulmonary Disease	pulmonary function tests tutorials (University of Iowa Virtual Hospital)	guide diagnosis and management
Type 2 Diabetes Mellitus	standards of care (American Diabetes Association)	develop a management plan
Back Pain	national guidelines (National Guidelines Clearinghouse)	determine if imaging is indicated
Preventive Medicine	clinical preventive services guide (Agency for Healthcare Research and Quality)	develop prevention plans for different age and gender patients

**Table 3:** Percentage of student completing checklist items

**Checklist Item**	**Student performance**				
	
	**FM (n = 19)**	**A/G (n = 23)**	**1^st ^(n = 24)**	**3^rd ^(n = 18)**	**ALL (n = 42)**
**Non-technology items (8)**					
Asked how long I would be staying	89%	100%	100%	89%	95%
Asked if I had any medical problems	84%	96%	96%	83%	90%
Asked if I take any medications	68%	83%	71%	83%	76%
Asked if I smoked	26%	35%	46%	11%	31%
Asked me about prior immunizations	89%	100%	92%	100%	95%
Discussed protection from insect bites	74%	78%	75%	78%	76%
Offered a tetanus shot	84%	83%	79%	89%	83%
Offered any other type of injection	74%	83%	71%	89%	79%
**Technology items (13)**					
Asked if I would be providing health care on my travels	21%	0%	8%	11%	10%
Asked if I would be working with animals on my travels	37%	57%	50%	44%	48%
Asked if I would be outdoors or have contact with mosquitoes on my travels	74%	74%	79%	72%	74%
Sat so that I could see the Internet information on the tablet screen	68%	74%	67%	78%	71%
Offered a choice of medication options	47%	48%	42%	50%	48%
Clearly outlined the recommendations for medications and shots	68%	91%	71%	100%	71%
Accessed information using the tablet PC during the encounter	84%	96%	83%	100%	90%
Accessed information using the palm pilot during the encounter	58%	65%	58%	67%	62%
Discussed drinking water and food safety	95%	96%	100%	89%	95%
Advised me not to swim in fresh water	26%	30%	29%	28%	29%
Offered medication to prevent malaria	100%	96%	96%	100%	98%
Told me about the potential interaction between my antacids and new medicine	37%	39%	29%	56%	38%
Gave recommendations on how to take the medication with my antacids or advised me to stop antacids	32%	22%	21%	39%	26%
**Sub-score technology items (of 13)**	7.5	7.9	7.3	8.3	7.7
**OVERALL SCORE (Of 23)**	13.4	14.4	13.6	14.6	14.0

FM (Family Medicine Clerkship); A/G (Ambulatory Medicine/Geriatrics Clerkship); 1^st ^(first clerkship); 3^rd ^(third clerkship); ALL (students in FM and A/G)

## References

[B1] Ely JW, Burch RJ, Vinson DC (1992). The information needs of family physicians: case-specific clinical questions.[see comment]. Journal of Family Practice 35(3):265-9,.

[B2] Fozi K, Teng CL, Krishnan R, Shajahan Y (2000). A study of clinical questions in primary care. Medical Journal of Malaysia 55(4):486-92,.

[B3] Ramos K, Linscheid R, Schafer S (2003). Real-time information-seeking behavior of residency physicians.[see comment]. Family Medicine 35(4):257-60,.

[B4] Gorman PN, Ash J, Wykoff L (1994). Can primary care physicians' questions be answered using the medical journal literature?[see comment]. Bulletin of the Medical Library Association 82(2):140-6,.

[B5] Ely JW, Osheroff JA, Ebell MH, Chambliss ML, Vinson DC, Stevermer JJ, Pifer EA (2002). Obstacles to answering doctors' questions about patient care with evidence: qualitative study. BMJ 324(7339):710,.

[B6] Mahoney JF, Cox M, Gwyther RE, O'Dell DV, Paulman PM, Kowlowitz V (2004). Evidence-based and population-based medicine: national implementation under the UME-21 project. Family Medicine 36 Suppl:S31-5,.

[B7] Bergus G, Vogelgesang S, Tansey J, Franklin E, Feld R (2004). Appraising and applying evidence about a diagnostic test during a performance-based assessment. BMC Medical Education 4(1):20,.

[B8] Weberschock TB, Ginn TC, Reinhold J, Strametz R, Krug D, Bergold M, Schulze J (2005). Change in knowledge and skills of Year 3 undergraduates in evidence-based medicine seminars. Medical Education 39(7):665-71,.

[B9] Davidson RA, Duerson M, Romrell L, Pauly R, Watson RT (2004). Evaluating evidence-based medicine skills during a performance-based examination. Academic Medicine 79(3):272-5,.

